# Sympatry Predicts Spot Pigmentation Patterns and Female Association Behavior in the Livebearing Fish *Poeciliopsis baenschi*

**DOI:** 10.1371/journal.pone.0170326

**Published:** 2017-01-20

**Authors:** Andrea J. Roth-Monzón, Laura E. Scott, Ashley A. Camargo, Eliza I. Clark, Eric E. Schott, Jerald B. Johnson

**Affiliations:** 1 Evolutionary Ecology Laboratories, Department of Biology, Brigham Young University, Provo, Utah, United States of America; 2 Monte L. Bean Life Science Museum, Brigham Young University, Provo, Utah, United States of America; University of Arkansas, UNITED STATES

## Abstract

In this study, we explored the possibility that differences in pigmentation patterns among populations of the fish *Poeciliopsis baenschi* were associated with the presence or absence of the closely related species *P*. *turneri*. If reproductive character displacement is responsible, spotting patterns in these two species should diverge in sympatry, but not allopatry. We predicted that female *P*. *baenschi* from sympatric sites should show a preference for associating with conspecifics vs. heterospecific males, but females from allopatric sites should show no such preferences. To evaluate these predictions, we compared spotting patterns and female association behaviors in populations of *P*. *baenschi* from Central Mexico. We found that both of our predictions were supported. *Poeciliopsis baenschi* that co-occured with *P*. *turneri* had spotting patterns significantly different than their counterparts from allopatric sites. Using a simultaneous choice test of video presentations of males, we also found that female *P*. *baenschi* from populations that co-occured with *P*. *turneri* spent significantly more time with males of their own species than with *P*. *turneri* males. In contrast, females from allopatric populations of *P*. *baenschi* showed no differences in the amount of time they spent with either conspecific or heterospecific males. Together, our results are consistent with the hypothesis that reproductive character displacement may be responsible for behavioral and spotting pattern differences in these populations of *P*. *baenschi*.

## Introduction

A variety of phenotypic cues can be used in species recognition, including visual, chemical, and auditory cues. When closely related species do co-occur, they often diverge from one another in species recognition traits relative to their conspecific counterparts that occur in allopatry. Such divergence helps minimize costly reproductive interactions between heterospecifics. This form of divergence is known as reproductive character displacement (hereafter referred to as RCD) [1–8].

A variety of selective pressures can favor RCD. Reproductive interactions between heterospecifics can result in a decrease in fitness, either by hybridization or through reproductive interference, wherein heterospecifics lower the efficacy of conspecific matings [1–18]. Hybrid offspring are potentially costly because they could be inviable, sterile, have dysfunctional genomes, or simply be less fit for their environment than either parent [4,6,19–23]. When hybridization does reduce fitness [15,24–28], individuals that decrease the chance of heterospecific matings will have a selective advantage. Even when hybridization does not occur, interacting with heterospecifics can have fitness costs by wasting time and energy or result in injuries [2,10,17,29–31].

Studies of RCD have frequently been conducted in systems where females actively choose males using a sexual display to attract females (e.g. [9,11,15]). However, in many species, female choice is more subtle, e.g. where males attempt to force copulations [32,33]. In such systems females can exert choice by varying the extent to which they resist mating attempts, or by simply spending more time in proximity to some males versus others [34–36]. Indeed, evidence thus far suggests that females play an important role in male choice in both displaying and in coercive systems [37]. In both cases a key indicator is which males the females choose to associate with. In studies of RCD, females preferentially associating with conspecific males can reduce the chance of hybridization and reproductive interference [3,38–41]. Theory thus predicts that this preferential association should be strongest in sympatric populations.

The livebearing fishes *Poeciliopsis baenschi* and *P*. *turneri* from central Mexico provide an excellent system to examine the effects of RCD in a coercive mating system. These two species are phenotypically similar, but differ most strikingly in the number and shape of melanin-based spots along the lateral flank of their body [42]. Differences in these spotting patterns appear to be consistent, regardless of environmental stimuli [43]. Interestingly, *P*. *baenschi* also show intraspecific variation for these traits, but little is known about the factors responsible. Several studies suggest that barring or spotting patterns can be used as visual cues in species recognition [44] or identifying potential mates [45–47]. Moreover, unlike other pigments, melanin is synthesized internally and can therefore be consistently displayed as a reliable signal for species recognition [48–50]. Finally, the *Poeciliopsis* species evaluated here are members of two distinct monophyletic clades that have come into secondary contact in western Mexico [51,52], providing a set of sympatric populations that can be compared to allopatric populations in adjacent river drainages.

Here, we ask if *P*. *baenschi* show phenotypic divergence in spotting patterns and association behaviors consistent with the hypothesis of reproductive character displacement. We compared these traits using a combination of field collections and laboratory experiments. We evaluated two predictions; 1) *P*. *baenschi* from sympatric populations (those that co-occur with *P*. *turneri*) should show greater divergence in spotting traits from *P*. *turneri* than those found in allopatry, and 2) female *P*. *baenschi* from sympatric populations should have a greater tendency to associate with conspecific males over heterospecific males when compared to female *P*. *baenschi* from allopatric populations. We found that both of these predictions were supported by our data.

## Materials and Methods

### Study System and Field Collections

**Fig 1:**
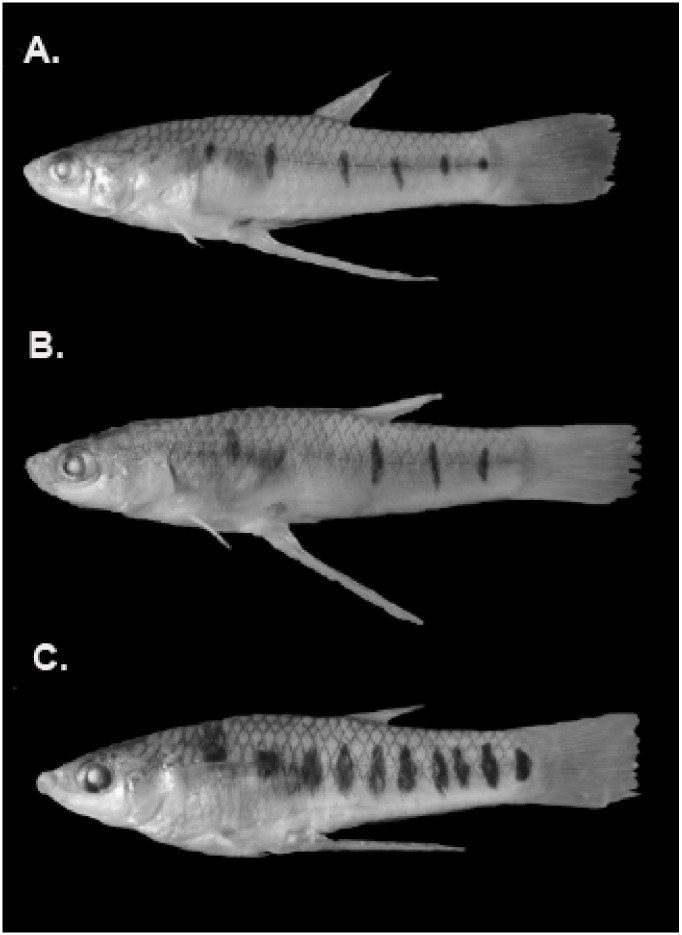
Photographs of a typical male from each of the three groups used in this study. **(A)** allopatric *P*. *baenschi* **(B)** sympatric *P*. *baenschi*, and **(C**) *P*. *turneri*.

**Fig 2:**
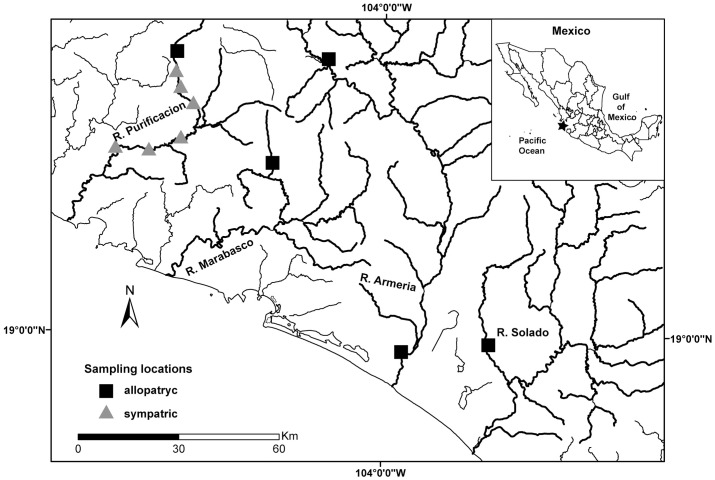
Map indicating locations of allopatric and sympatric collection sites. Numbers correspond with the collection sites as denoted in Table 1. Rivers sampled are bold with corresponding name near them. Star in inset Mexico map indicates area for the full map. Shapes for map construction were obtained from Instituto Nacional de Estadística y Geografía (INEGI).

*Poeciliopsis baenschi* and *P*. *turneri* (Fig 1) are members of the family Poecilidae, a group of livebearing fishes that give birth to free-swimming, precocial young. Both species are narrowly distributed along the central-western coast of Mexico (Fig 2). In some locations, *P*. *baenschi* co-occurs with *P*. *turneri*, and in other locations it occurs alone. Populations of *P*. *turneri* do not occur without *P*. *baenschi*.

We collected *P*. *baenschi* and *P*. *turneri* from 11 localities in western Mexico during May and June 2007 (Fig 2, Table 1). Our samples included six localities where *P*. *baenschi* co-occurs with *P*. *turneri* and five localities where *P*. *baenschi* occurs alone (Fig 2). All sympatric localities were taken from the same drainage system where these species have come into secondary contact [53]. Each sympatric collection was made at least 3.5 km apart to ensure these were independent replicates. Although we do not know the extent to which gene flow occurs among these populations, our analyses of spotting patterns did not show a clinal gradient (S1 Fig), suggesting that each sympatric sampling locality is distinct.

**Table 1:** Locality information for populations of *P*. *baenschi* and *P*. *turneri* used in this study.

Habitat	Collection Site	ID	GPS coordinates	
			Latitude	Longitude
Sympatric	1	M07-01	19.701	-104.598
	2	M07-29	19.676	-104.577
	**3**	**M07-02**	**19.622**	**-104.548**
	4	M07-03	19.529	-104.582
	5	M06-11	19.495	-104.672
	6	M07-31	19.501	-104.767
Allopatric	**7**	**M07-28**	**19.746**	**-104.552**
	8	M07-04	19.462	-104.319
	9	M07-26	19.745	-104.158
	10	M07-17	18.956	-103.945
	**11**	**M07-19**	**18.977**	**-103.698**

In sympatric habitats, populations of both *P*. *baenschi* and *P*. *turneri* co-occur; in allopatric habitats, only *P*. *baenschi* are found. Fish from all collection sites were used in quantifying spotting patterns. Fish from the bolded collection sites were used in the behavioral assay.

All fish were collected with a hand-held seine net (1.3 m x 5 m; 8 mm mesh size). Fish used for the spotting pattern analysis were euthanized in the field, preserved in ethanol, and transported to the laboratory for data collection. Live fish used in the behavioral analysis were collected from three locations: site three (sympatric); and sites seven and 11 (allopatric; see Fig 2; Table 1). Live fish were transported to the laboratory at Brigham Young University where they were housed in 20 gallon tanks at 22°C, a temperature typical for these fishes in the wild. Fish were isolated by population, fed twice daily, and kept on a 12:12 hour LD cycle. The Institutional Animal Care and Use Committee at Brigham Young University approved the use of live fish for this study (IACUC protocol 06–0104) and all guidelines and recommendations in this protocol were strictly followed.

### Quantifying Spots

We collected spot data from ethanol-preserved samples. Although ethanol can diminish the intensity of melanin spots, we had no difficulty measuring the shape or number of spots in our samples. We quantified spotting patterns by measuring two primary characteristics of spots of reproductively mature males; (1) the number of spots; and (2) the total pigmented area of spots. Our focus on males was to complement the female association tests described below. Previous work suggests that spotting patterns can sometimes vary between different sides of the same fish [43]. However, we found that asymmetry in spot size and color between sides was low compared to differences between populations and species, and that neither side had an inherent bias in number or shape of spots (S2 Fig). Hence, we collected all of our data from the left side of each fish. We examined 98 specimens of *P*. *baenschi* from allopatric sites, 111 specimens of *P*. *baenschi* from sympatric sites, and 67 specimens of *P*. *turneri* from sympatric sites. From these samples, we quantified the number and total area of spots using ImageJ 1.41 (http://rsbweb.nih.gov/ij/). The spots of these species were conspicuous allowing us to count the total number of spots on each fish by eye. We measured the total area of spots covering the side of the fish bounded by the opening of the operculum at the anterior margin and the end of the vertebral column at the posterior margin. We quantified area (in mm^2^) by transforming each image to black and white and classifying pixels as pigmented or non-pigmented using a color threshold between 30 and 50.

### Behavioral Assays

Our behavioral assay allowed *P*. *baenschi* females from allopatric and sympatric locations to choose between males of their own species versus *P*. *turneri* males. We used a dichotomous-choice test with a video playback system [48,51,54,55] to determine if *P*. *baenschi* females derived from sympatric and allopatric sites showed a difference in their association times with conspecific versus heterospecific males.

To measure association time, a single *P*. *baenschi* female was simultaneously presented with two stimulus videos of males following previously published methods [56]. In brief, we created stimulus videos using a composite of video images of three males from each of our three focal populations (*P*. *baenschi* in sympatry, *P*. *baenschi* in allopatry, and *P*. *turneri*). The males used to create the stimulus tapes were of similar size (within 2 mm) and were typical in terms of spotting patterns for each population. We conservatively chose a 2 mm size difference threshold because it is smaller than the 3 mm difference shown to be necessary to have an effect on mate choice in similar studies of poeciliid fish mating preferences [57–59]. In addition, males used to make the videos were chosen to be as similar as possible, with the primary difference being the number and character of spots. The composite video provided a 10 minute looped segment of a single male swimming back and forth across the screen. Males in these looped videos did not show any courtship behavior, although the swimming behavior was typical of *Poeciliopsis* males that approach females from the side or behind prior to mating. Hence, females were simply given an opportunity to spend time with one video male or the other. Previous observations in other poeciliid fishes has shown that the male that a female associates with is frequently a good indicator of male mating success [56,60–63]. We also made control videos showing only the background with no stimulus.

We conducted a total of 10 trials for sympatric females and 11 trials for allopatric females, sample sizes sufficient to detect differences in association time in our study (see below). Females included in the study were separated from males for at least two week before starting the trials [64–66]. In each trial, a female was introduced into the tank and allowed to acclimate for 10 minutes while empty tank control videos were shown on monitors abutting the opposite sides of the test tank. Following the 10-minute acclimation period, we started the 10-minute male stimulus videos. *Poeciliopsis baenschi* females were presented with a choice between a conspecific and heterospecific male. To control for side preferences, we randomly assigned the side to which the males were presented. A video camera placed one meter from the front glass recorded each trial. All recording was done remotely from an adjacent room. Association time was defined as the amount of time a female spent in the third of the tank closest to the stimulus video [67].

### Statistical Analyses

We compared spotting patterns between groups using a general linear model framework. The number of spots and total area of spots were both analyzed by analysis of covariance (ANCOVA). In each model, we tested for differences between allopatric *P*. *baenschi*, sympatric *P*. *baenschi*, and sympatric *P*. *turneri*. The number of spots and total area of spots can covary with fish body size because larger fish have larger spots. Hence, we included “area of fish” as a covariate. We quantified “area of fish” by outlining the fish body in ImageJ over the same area for which spot pigment was measured. To meet the assumptions of the statistical models, ‘number of spots’ was square root transformed and ‘area of fish’ was natural log transformed. Our results were the same regardless of whether or not the data were transformed; hence, for ease of interpretation we present non-transformed results. The interaction term between the “groups” (allopatric *P*. *baenschi*, sympatric *P*. *baenschi*, and sympatric *P*. *turneri*) and “area of fish” was also included in each model to determine if spotting patterns changed among groups as a function of body size. Finally, we tested for differences in the two spot traits between sympatric and allopatric populations of *P*. *baenschi* (excluding *P*. *turneri*) using a one-way analysis of covariance (ANCOVA). These ANCOVA models used the same variables as those described above.

To analyze the association behavior data, we used a one-way analysis of variance (ANOVA). We compared the amount of time females spent associating with a conspecific male versus time spent with the heterospecific *P*. *turneri* male. Because females had the option of not interacting by remaining in the center of the tank, we treated the amount of time females spent with each male as an independent measure [56,62,68]. This statistical test was performed separately for the sympatric population and the allopatric population because we wanted to know if each population differed in their association with conspecifics versus heterospecifics. We also tested for a tank side-bias by comparing the amount of time spent on each side of the tank during our control treatments when the control videos were presented. In total, we present the results for four separate tests.

Statistical significance was evaluated at the P < 0.05 level. All statistical tests were conducted in R [69]. We report the least square means and standard error for the number of spots, total area of spots and association time from the behavioral assays in the results. For the number and total area of spots, the least square means were adjusted for the covariate (the differences in size of the groups), thus allowing us to compare the differences in number of spot between groups regardless of the differences in body size among individuals between the groups. For the behavioral data, we used least square means to account for differences in the number of replicates for each of the treatments (10 sympatric vs. 11 allopatric).

## Results

### Spotting Pattern

**Fig 3:**
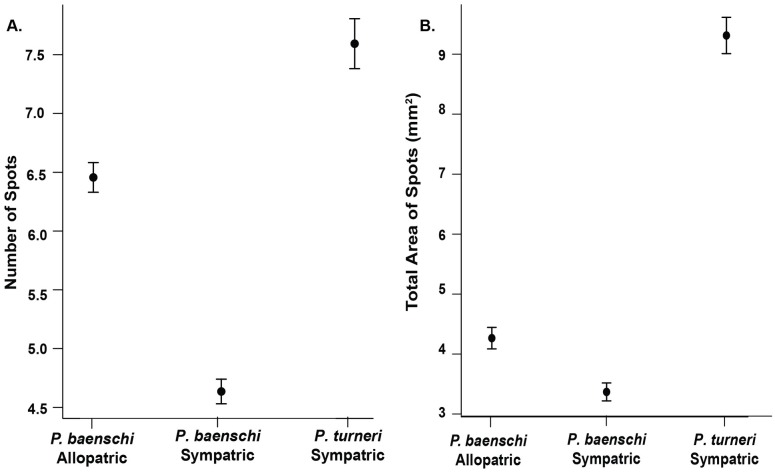
Comparisons of number of spots and total area of spots for allopatric *P*. *baenschi*, sympatric *P*. *baenschi*, and *P*. *turneri*. **(A)** Comparisons of number of spots per lateral flank for sympatric *P*. *baenschi*, allopatric *P*. *baenschi*, and *P*. *turneri*. **(B)** Comparisons of total area of spots per lateral flank of each fish for sympatric *P*. *baenschi*, allopatric *P*. *baenschi*, and *P*. *turneri*. Values presented are least square means from a general linear model analyses (± 1 SE).

**Fig 4:**
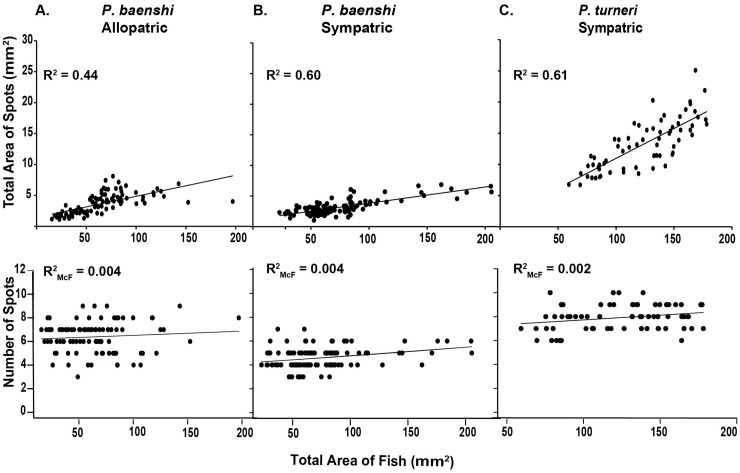
Relationship between total area of spots and number of spots with total area of fish (mm^2^) for allopatric *P*. *baenschi*, sympatric *P*. *baenschi*, and *P*. *turneri*. **(A)** allopatric *P*. *baenschi*, **(B)** sympatric *P*. *baenschi* and **(C)** *P*. *turneri*.

Allopatric *P*. *baenschi*, sympatric *P*. *baenschi* and *P*. *turneri* differed significantly from one another in their spotting patterns (Table 2; Fig 3). Consistent with our predictions, we found that sympatric populations were more divergent from *P*. *turneri* than were allopatric populations for both the number of spots and total pigmented area (Fig 3). *Poeciliopsis turneri* had the greatest number of spots (mean ± 1 SE, 7.58 ± 0.21), sympatric populations of *P*. *baenschi* had the fewest number of spots (4.64 ± 0.10), and allopatric populations of *P*. *baenschi* had spot numbers intermediate between the other two groups (6.45 ± 0.13; Fig 3A). Total area of spots varied significantly between the three groups. *Poeciliopsis turneri* had the greatest area (mm^2^) of spots (9.29 ± 0.30), sympatric populations of *P*. *baenschi* had the smallest area of spots (3.36 ± 0.15), and allopatric populations of *P*. *baenschi* were intermediate, although closer to sympatric *P*.*baenschi* than *P*. *turneri* (4.26 ± 0.18; Fig 3B). We also found a significant interaction between group and area of fish (fish size) for both the number of spots and total area of spots (Table 2; Fig 4), indicating that spot area and number of spots scaled with body size, but did so in different ways for each of the groups. At smaller body sizes, the total area of spots was similar between the three groups, but at larger body sizes, *P*. *turneri* had a significantly greater area of spots than both sympatric and allopatric populations of *P*. *baenschi* (Table 2; Fig 4). Area of fish was not a good predictor of number spots, as shown by the low goodness of fit of the models (R^2^_McF_ = 0.004, 0.004, 0.002; Fig 4; [70]).

**Table 2:** Analysis of covariance results comparing spotting pattern traits between the three groups (see text).

Response Variable	Effect	*F*	*df*	P
Total number of spots per fish	Group	209.96	2, 270	<0.001
	Area of fish	9.94	1, 270	0.002
	Area of fish X Group	0.43	2, 270	0.69
Total area of spots per fish (mm^2^)	Group	1064.22	2, 270	<0.001
	Area of fish	268.06	1, 270	<0.001
	Area of fish X Group	52.34	2, 270	<0.001

The covariate was area of fish, an estimate of fish size.

Our second set of analyses focused on the comparison between sympatric and allopatric populations of *P*. *baenschi*. We found that sympatric populations of *P*. *baenschi* had significantly fewer spots (4.55 ± 0.10) than allopatric populations (6.41 ± 0.11; F_1,205_ = 145.45, P < 0.001). We also found that the total area of spots was smaller in sympatric populations (3.01 ± 0.10 mm^2^) than in allopatric populations (3.81 ± 0.11 mm^2^; F_1,205_ = 11.17, P < 0.001). Interestingly, total spot area and number of spots were only weakly correlated (R = 0.24, P < 0.001) in *P*. *baenschi*, suggesting the potential for these traits to function somewhat independently.

### Behavioral Assay

**Fig 5:**
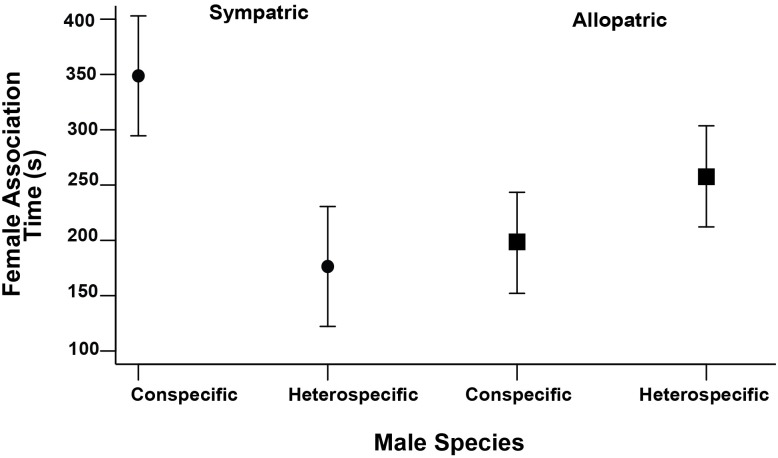
Results from the behavioral assay comparing *P*. *baenschi* female association time with a conspecific (*P*. *baenschi*) or heterospecific (*P*. *turneri*) male. Circles refer to sympatric *P*. *baenschi* and squares refer to allopatric *P*. *baenschi*. Values presented are least square means from a general linear model (± 1 SE) for the amount of time females spent near the indicated side of the tank.

When given the choice between heterospecific and conspecific males, sympatric *P*. *baenschi* females showed a clear association preference for conspecific males in terms of time spent in seconds with each type of male (conspecific: 348.72 ± 54.18 *s*; heterospecific: 176.4 ± 54.18 *s*; ANOVA F_1,18_ = 5.06, P = 0.04; Fig 5). In contrast, allopatric *P*. *baenschi* females showed no significant difference in their association time between their own species versus *P*. *turneri* (conspecific: 197.77 ± 45.69 *s*; heterospecific: 257.87 ± 45.69 *s;* ANOVA F_1,20_ = 0.86, P = 0.36; Fig 5). To check whether our non-significant results were due to lack of statistical power, we conducted a post hoc power analysis using G Power 3 [71] and found that for the effect size observed in the present study (d = 0.198) with an alpha of 0.05, a sample of approximately 202 would be needed to obtain statistical power at the recommended 0.80 level [72]. Moreover, our controls revealed no evidence for a tank side effect because individuals were equally likely to spend time on either side of the tank in the absence of the male stimulus (sympatric female, right side: 205.78 ± 41.43 *s*; left side: 293.78 ± 41.43 *s*; ANOVA F_1,18_ = 2.23, P = 0.15 and allopatric female, right side: 227.98 ± 43.57 *s*; left side: 190.05 ± 43.57 *s*; ANOVA F_1,20_ = 0.38, P = 0.55). Again, power analysis revealed that for the effect size observed here for both sympatric (d = 0.334) and allopatric females (d = 0.131) with an alpha of 0.05, a sample of approximately 74 and 458 would be needed to obtain statistical power at the recommended 0.80 level [72]. All power tests showed that to achieve a level of 0.80, sample sizes that are prohibitively large for most behavioral studies are requiered. Given that our non-significant results greatly overlapped, it is reasonable to conclude that no differences were observed.

## Discussion

Our results are consistent with the reproductive character displacement hypothesis. Spotting patterns differed more between *Poeciliopsis baenschi* and *P*. *turneri* where these species co-occur than when *P*. *baenschi* occurs alone. Also, *P*. *baenschi* females from sympatric sites showed preferences for males of their own species, but no such preference was observed in *P*. *baenschi* from allopatric sites. Why selection should favor reproductive character displacement in these fishes? Two plausible explanations are reinforcement and reproductive interference.

Although some species in the genus *Poeciliopsis* are known to hybridize [73,74], most taxa in the genus maintain distinct species boundaries. To date, no evidence of hybridization between *P*. *baenschi* and *P*. *turneri* exists, so we cannot rule out the possibility that reinforcement has led to our observed differences, but we consider it unlikely. In contrast, sexual interference is a plausible explanation given the overall phenotypic similarity between these two taxa and the similar male mating tactics of forcing copulations. Under these circumstances, mistaken mating attempts by males can be costly to females [75–79]. Costs such as energy investment to avoid males or actual injuries caused by males have caused females to change their association behavior to avoid harassment in closely related species [75–79]. Our data are consistent with this explanation. It is possible that the female association preferences observed here are not completely related to spotting patterns, but could be explained by confounding traits such as shape. However, we consider this unlikely given that we matched stimulus males to control for such differences. This matching accounted for coloration and male size traits that can influence visual preference in poeciliid female [9,62,80–82].

Even though our data are consistent with an explanation of RCD, other factors could contribute to the observed patterns including confounding ecological variables [4,12,83] and differential fusion, where species that come into secondary contact either fuse or are maintained depending on the strength of mating discrimination present before secondary contact.

Ecological variables that affect the presence or discrimination of a phenotype could lead to changes in mating cues or association preference [18,82,84–86]. It is possible that the relationship between spot number and species co-occurrence is driven by an unidentified common ecological factor. For example, both predation pressure and resource availability have been shown to affect pigmentation in poeciliid fishes [73,74,87–93]. Predation can cause either an increase or decrease in pigmentation, depending on the degree to which it affects the conspicuousness of an individual. Unfortunately, there are no known differences in predation pressure among our collecting localities [94]. Environmental resources can also affect fish pigmentation where pigments are directly obtained from the diet [48,54,55,75–79]. Unfortunately, this is not the case with melanin, a pigment that is not diet derived but is instead synthesized internally. Previous work [94] has demonstrated that the local environments of *P*. *baenschi* evaluated here—both sympatric and allopatric—do not differ in resource availability.

The hypothesis of differential fusion suggests that when species come in secondary contact, they fuse or persist as distinct species depending on the strength of mating discrimination that existed in allopatry [12]. Our results show that allopatric *P*. *baenschi* females had no association preference and thus had no effect on male mating potential. Therefore differential fusion is an unlikely explanation. Our best explanation is that the presence or absence of heterospecific *P*. *turneri* has influenced spotting patterns and female association preferences in *P*. *baenschi*.

In conclusion, our results are consistent with the explanation that species recognition behavior has evolved in sympatric sites where there are potential fitness risks caused by the presence of a heterospecific, but is absent in allopatric sites where there are no such risks. Consistent with our findings, several other studies suggest that barring or spotting patterns on fish can be used as visual cues in species recognition [44]. Differences in spotting patterns and species association behaviors observed here suggest that sympatric populations of *P*. *baenschi* express phenotypes that reduce the possibility of reproductive interference. Whether these differences are genetically based or environmentally induced remains unknown. More research is needed to directly determine what fitness benefits sympatric populations of *P*. *baenschi* achieve in their habitat relative to allopatric populations. Nonetheless, our study points to the presence of the closely related fish *P*. *turneri* as an important factor for shaping phenotypic divergence in both spotting patterns and association behavior among *P*. *baenschi* populations.

## Supporting Information

S1 Fig Boxplot of both number of spots and total area of spots for sympatric *P*. *baenschi* sampled localities. (TIF)

S2 Fig Photographs of a typical male from each of the three groups used in this study from both left and right side. **(A)** allopatric *P*. *baenschi* **(B)** sympatric *P*. *baenschi*, and **(C**) *P*. *turneri*.
(TIF)
