# Supplementary figures and images for: Sympatry Predicts Spot Pigmentation Patterns and Female Association Behavior in the Livebearing Fish Poeciliopsis baenschi
Source: PLoS One. 2017 Jan 20;12(1):e0170326. doi: 10.1371/journal.pone.0170326 (PMC5249170; doi:10.1371/journal.pone.0170326)

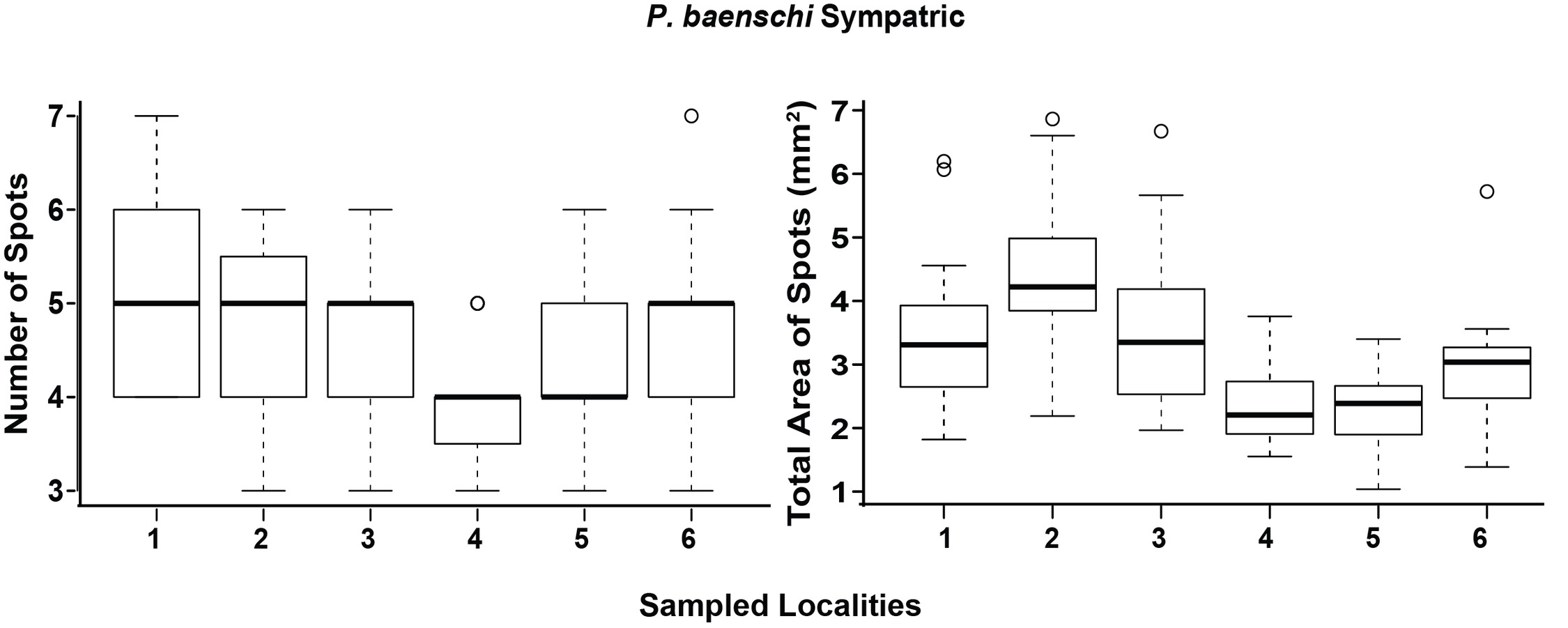

Supplement: S1 Fig — (TIF) [file pone.0170326.s001.tif]

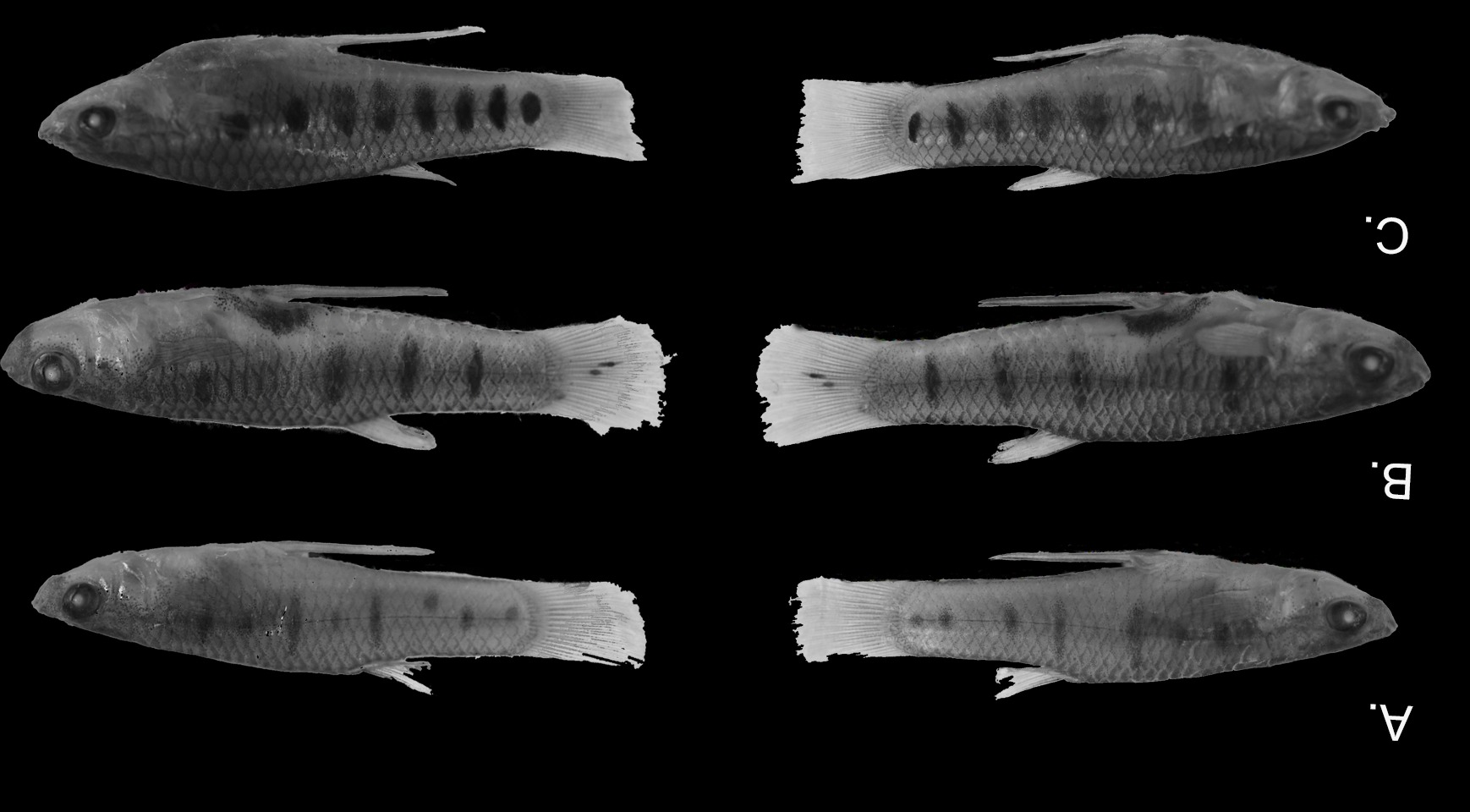

Supplement: S2 Fig — (A) allopatric P. baenschi (B) sympatric P. baenschi, and (C) P. turneri. (TIF) [file pone.0170326.s002.tif]
